# Effects of different water management and fertilizer methods on soil temperature, radiation and rice growth

**DOI:** 10.1038/s41598-022-20764-w

**Published:** 2022-09-29

**Authors:** Ennan Zheng, Jianyu Hu, Yinhao Zhu, Tianyu Xu

**Affiliations:** School of Hydraulic and Electric Power, Heilongjiang University, Harbin, 150080 China

**Keywords:** Ecology, Plant sciences

## Abstract

In recent decades, the application of organic fertilizer to agricultural soils has attracted wide attention. However, few studies have carefully explored the effects of humic acid fertilizer on soil temperature, radiation, and the physiology of plant leaves, especially when coupled with different irrigation methods. To provide a better growing environment for crops and explore the best regulation method of humic acid fertilizer and irrigation in the farmland soil environment on the Songnen Plain, China, through field experiments, we selected rice as the test crop and applied humic acid fertilizer to the soil with different irrigation methods. The effects of different humic acid fertilizers and irrigation methods on the soil temperature and radiation changes during different growth stages were examined, and the subtle differences in agronomic and fluorescence characteristics in different growth stages of rice plants were compared. The results showed that the soil temperature was not significantly different among all the treatments. However, radiation interception was obviously different, and the best value was observed in the CT5 treatment. The fluorescence indices and leaf chlorophyll relative content (*SPAD*) differed with the change in humic acid fertilizer application and irrigation methods. At the jointing and heading stages, the *F*_v_ */F*_m_ values of the CT5, FT5 and WT5 treatments were larger than those of the other treatments, and the best value was recorded in the CT5 treatment. The differences in *NPQ* at these two stages were significant, and the *NPQ* in the CT5 treatment was significantly higher than that in the other treatments (*P* < 0.05). In general, the *Q*_P_ under control irrigation was greater than that under flood and wet irrigation (*P* < 0.05). Moreover, there were no significant differences among the gradients under the different humic acid fertilizer application methods in terms of *Q*_P_ (*P* > 0.05). Additionally, *SPAD* values were higher under the CT5 and FT5 treatments.

## Introduction

In the past 30 years, farmland has been rapidly developed and used, with a large number of fertilizers and pesticides applied to the soil, which not only accelerates the degradation of land^1^ but also causes ecological environmental pollution^2–4^, leading to a decrease in agricultural product quality and production efficiency. Therefore, agricultural production capacity and sustainable development face severe challenges^5–7^. The core problem of farmland soil degradation is the decline in the quantity and quality of organic matter; therefore, guaranteeing the stability of organic matter in farmland soil is one of the key factors. There is also an important factor of ensuring the safety of farmland soil, that is, water resources, in which the decline of the groundwater level is accelerating on the Songnen Plain, China^8–10^; therefore, it is necessary to develop water-saving irrigation in this region to promote water resources and protect the safety of farmland soil.

By reviewing the literature, we found that most experiments have only studied the effects of humic acid fertilizer on crops and farmland soil environments. For example, Li^11^ found that applying humic acid fertilizer to farmland soil could not only optimize the farmland soil structure and increase the utilization rate of fertilizers but could also promote the growth of crops. Meng^12^ applied humic acid fertilizer to saline-alkali soil, and the results indicated that it could enhance salt leaching and inhibit nitrogen loss in the topsoil. The results of Yan^13^ and Li^14^ showed that farmland soil nutrient contents, yield and quality of crops were increased by the application of humic acid fertilizer.

Meng^15^ showed that because of the application of humic acid fertilizer, farmland soil microaggregates increased by 77.59–125.58%. In addition, l salehi^16^ considered that the different rates of humic acid fertilizer could improve morphological and biochemical traits in nasturtium flowers. Ren^17^ showed that humic acid fertilizer could reduce farmland soil pH and increase organic matter under certain circumstances thus enhancing growth and increasing the yield of rice. Gu^18^ and Chen^19^ concluded that humic acid fertilizer could improve farmland soil physical and chemical properties, reduce compaction, increase farmland soil fertility, stimulate plant growth and increase plant stress resistance. Liu^20^ and Qian^21^ found that plant height, stem diameter, ratio of root to shoot, and root activities were significantly increased by the addition of humic acid fertilizer. In summary, studies examining the influences of different humic acid fertilizers and irrigation techniques on crops have been minimal, especially in paddy fields. Therefore, on the basis of these studies, we explored the effects of different humic acid fertilizers and irrigation methods on paddy fields. To maintain farmland soil tillage and the health of the growing environment, we determined the best regulation methods of humic acid fertilizer and irrigation for farmland growing environments that are suitable for the Songnen Plain through field experiments.

We selected humic acid fertilizer and irrigation methods for a field rice-planting experiment, and we made the following hypothesis: (1) whether different humic acid fertilizers and irrigation methods could affect temperature and radiation interception; (2) whether an appropriate amount of humic acid fertilizer and irrigation methods would promote rice growth and have a positive impact on agronomic traits; and (3) whether different humic acid fertilizers and irrigation methods on the fluorescence of plants were diverse.

## Materials and methods

### General description of the experimental area

**Figure 1:**
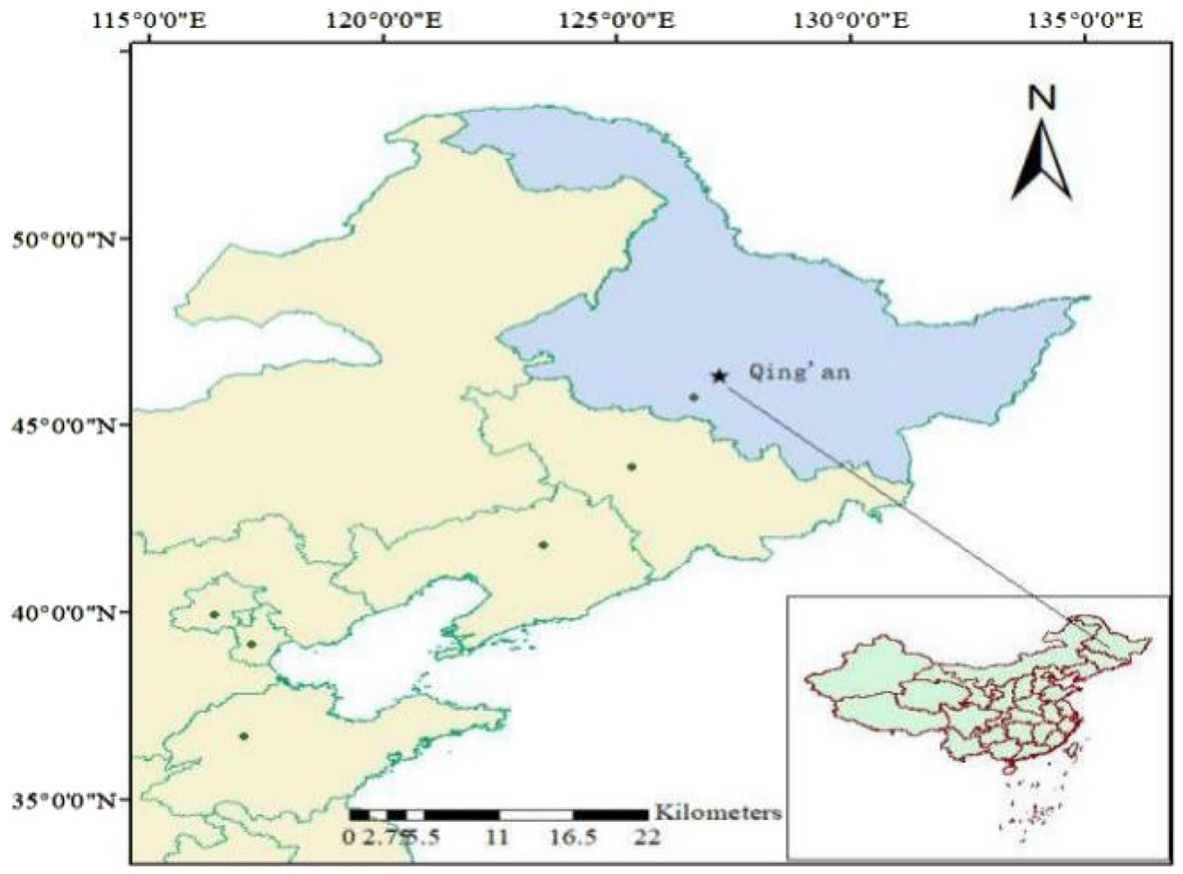
Location of the study area. The map and inset map in this image were drawn by the authors using ArcGIS software. The software version used was ArcGIS software v.10.2, and its URL is http://www.esri.com/.

The experiment was performed for two years at the National Key Irrigation Experimental Station located on the Songnen Plain in Heping town, Qing'an County, Suihua, Heilongjiang, China, with a geographical location of 45° 63′ N and 125° 44′ E at an elevation of 450 m above sea level (Fig. 1). This region consists of plain topography and has a semiarid cold temperate continental monsoon climate, i.e., a typical cold region with a black soil distribution area. The average annual temperature is 2.5 °C, the average annual precipitation is 550 mm, the precipitation is concentrated from June to September of each year, and the average annual surface evaporation is 750 mm. The growth period of crops is 156–171 days, and there is a frost-free period of approximately 128 days year^−1^^22^. The soil at the study site is albic paddy soil with a mean bulk density of 1.01 g/cm^3^ and a porosity of 61.8% prevails. The basic physicochemical properties of the soil were as follows: the mass ratio of organic matter was 41.8 g/kg, pH value was 6.45, total nitrogen mass ratio was 15.06 g/kg, total phosphorus mass ratio was 15.23 g/kg, total potassium mass ratio was 20.11 g/kg, mass ratio of alkaline hydrolysis nitrogen was 198.29 mg/kg, available phosphorus mass ratio was 36.22 mg/kg and available potassium mass ratio was 112.06 mg/kg.

### Humic acid fertilizer

**Figure 2:**
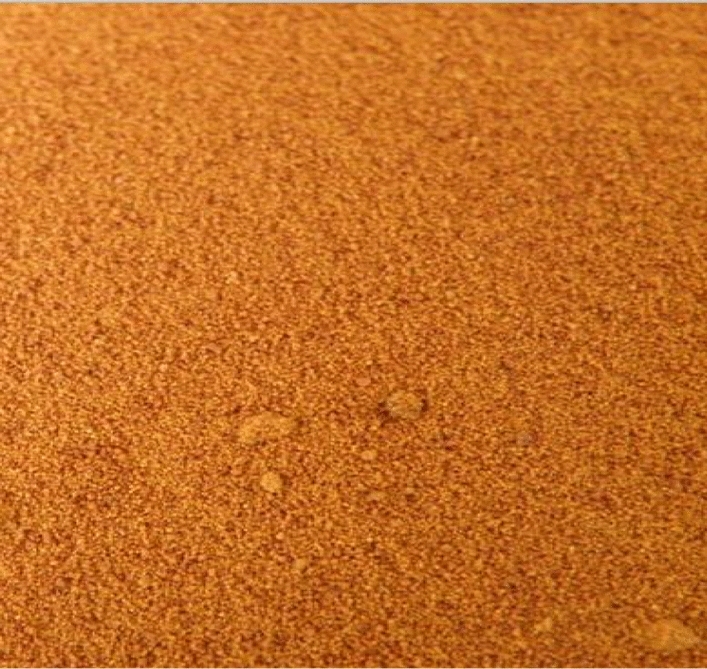
Humic acid fertilizer in powder form.

Humic acid fertilizer was produced by Yunnan Kunming Grey Environmental Protection Engineering Co., Ltd., China (Fig. 2). The organic matter was ≥ 61.4%, and the total nutrients (nitrogen, phosphorus and potassium) were ≥ 18.23%, of which N ≥ 3.63%, P_2_O_5_ ≥ 2.03%, and K_2_O ≥ 12.57%. The moisture content was ≤ 2.51%, the pH value was 5.7, the worm egg mortality rate was ≥ 95%, and the amount of faecal colibacillosis was ≤ 3%. The fertilizer contained numerous elements necessary for plants. The contents of harmful elements, including arsenic, mercury, lead, cadmium and chromium, were ≤ 2.8%, 0.01%, 7.6%, 0.1% and 4.7%, respectively; these were lower than the test standard.

### Experimental design and observation methods

#### Irrigation

In this experiment, three irrigation practices, namely, control irrigation (C), wet irrigation (W) and flood irrigation (F), were designed (Table 1).

**Table 1:** Different irrigation methods.

Irrigation practices	Regreen stage	Early tillering	Middle tillering	Later tillering	Jointing stage	Heading stage	Ripening stage	Yellow stage
C	0–30 mm	85%*θs*–*θs*	85%*θs*–*θs*	Field drying	85%*θs*–*θs*	85%*θs*–*θs*	85%*θs*–*θs*	Drainage
W	0–30 mm	0–30 mm	0–30 mm	Field drying	0–30 mm	0–30 mm	0–30 mm	Drainage
F	10–50 mm	10–50 mm	10–30 mm	Field drying	10–50 mm	10–50 mm	10–30 mm	Drainage

*θ*_*s*_ is the saturated soil water content; before “–” is the lower limit of irrigation and after “–” is the upper limit of irrigation; C represents control irrigation; W represents wet irrigation; F represents flood irrigation; the numbers represent water layer depth; and the percentage represents percent content of saturated soil water content.

Control irrigation (C) of rice had no water layer in the rest of the growing stages, except for the shallow water layer at the regreen stage of rice, which was maintained at 0–30 mm, and the natural dryness in the yellow stage. The irrigation time and irrigation quota were determined by the root soil moisture content as the control index. The upper limit of irrigation was the saturated moisture content of the soil, the lower limit of soil moisture at each growth stage was the percentage of saturated moisture content, and the TPIME-PICO64/32 soil moisture analyser was used to determine the soil moisture content at 7:00 a.m. and 18:00 p.m., respectively. When the soil moisture content was close to or lower than the lower limit of irrigation, artificial irrigation occurred until the upper irrigation limit was reached. The soil moisture content was maintained between the upper irrigation limit and the lower irrigation limit of the corresponding fertility stage. Under the wet irrigation (W) and flood irrigation (F) conditions, it was necessary to read the depth of the water layer through bricks and a vertical ruler embedded in the field before and after 8:00 am every day to determine if irrigation was needed. If irrigation was needed, then the water metre was recorded before and after each irrigation. The difference between before and after was the amount of irrigation^23^.

#### Fertilization

In our research, five fertilization methods were applied, as shown in Table 2. In this experiment, the rice cultivar “Suijing No. 18” was selected. Urea and humic acid fertilizer were applied according to the proportion of base fertilizer:tillering fertilizer:heading fertilizer (5:3:2). The amounts of phosphorus and potassium fertilizers were the same for all treatments, and P_2_O_5_ (45 kg ha^−1^) and K_2_O (80 kg ha^−1^) were used. Phosphorus was applied once as a basal application. Potassium fertilizer was applied twice: once as a basal fertilizer and at 8.5 leaf age (panicle primordium differentiation stage) at a 1:1 ratio^22^.

**Table 2:** The fertilizer methods.

Treatments	Pure nitrogen N (kg∙ha^−1^)	Humic acid fertilizer (kg∙ha^−1^)	P_2_O_5_ (kg∙ha^−1^)	K_2_O (kg∙ha^−1^)
T1	110	0	45	80
T2	77	450	45	80
T3	55	750	45	80
T4	33	1050	45	80
T5	0	1500	45	80

This study was performed with a randomized complete block design with three replications. Three irrigation practices and five fertilizer methods were applied, for a total of 15 treatments as follows: CT1, CT2, CT3, CT4, CT5; WT1, WT2, WT3, WT4, WT5; FT1, FT2, FT3, FT4, and FT5 (C, W, and F represent control irrigation, wet irrigation, and flood irrigation; T represents fertilizer treatment).

### Measurements of the samples

A soil temperature sensor (HZTJ1-1) was buried in each experimental plot to monitor the temperature of each soil layer (5 cm, 10 cm, 15 cm, 20 cm and 25 cm depth). The transmission of photosynthetically active radiation was measured from 11:00 to 13:00 by using a SunScan Canopy Analysis System (Delta T Devices, Ltd., Cambridge, UK), and data during the crop-growing season were recorded every day^24^.

Plant measurements were taken during the periods of tillering to ripening on days with no wind and good light. The fluorescence parameters were measured by a portable fluorescence measurement system (Li-6400XT, America). The detection light intensity was 1500 μmol m^−2^ s^−1^, and the saturated pulsed light intensity was 7200 μmolm^−2^ s^−1^. The functional leaves were dark adapted for 30 min, and then the maximum photosynthetic efficiency of PSII (*F*_v_*/F*_m_) was measured. Photochemical quenching (*Q*_P_) and nonphotochemical quenching (*NPQ*) were measured with natural light. Simultaneously, the leaf chlorophyll relative content (*SPAD*) was monitored using *SPAD* 502 (Konica Minolta, Inc., Tokyo, Japan). For plant agronomic characteristics, the distance from the stem base to the stem tip was measured with a straight ruler to quantify plant height^24^.

### Statistical analysis

Experimental data obtained for different parameters were analysed statistically using the analysis of variance technique as applicable to randomized complete block design. Duncan's multiple range test was employed to assess differences between the treatment means at a 5% probability level. All statistical analyses were performed using SPSS 22.0 for Windows^24^.

### Ethics approval

Experimental research and field studies on plants, including the collection of plant material, comply with relevant institutional, national, and international guidelines and legislation. We had appropriate permissions/licences to perform the experiment in the study area.

## Results

### Variation in soil temperature

In this experiment, the temperature data for 5–25 cm of the soil plough layer were analysed. The average temperatures of the soil layer during the entire growth period of the plant are shown in Table 3. Across all treatments, the soil temperature showed the same trend, which was generally divided into two parts. The first part, from 5 to 15 cm, was the soil surface in which the soil temperature gradually decreased, and the decrease in temperature was approximately 3–4 °C. In the second part, from 15 to 25 cm, the soil temperature rose slowly and continued to increase, and the temperature increase was below 1 °C. Under the different humic acid fertilizer and irrigation method conditions, the temperature differences from 15 to 25 cm were smaller than those from 5 to 15 cm. The surface soil temperature (5 cm and 10 cm) under the five humic acid fertilizer application methods showed a general trend of change in the order T2 > T3 > T1 > T4 > T5, while under the three irrigation conditions, the general trend showed the order C > W > F. Based on the above data, among all the different humic acid fertilizer and irrigation methods, although there were differences, we did not find statistical significance at the 5% probability (*P* > 0.05). It could be concluded that the humic acid fertilizer application with different irrigation methods was not beneficial for the maintenance of soil temperature.

**Table 3:** The average temperatures of the soil layer during the entire growth period in different treatments (°C).

Soil layer	First year					Second year				
	Treatments					Treatments				
	CT1	CT2	CT3	CT4	CT5	CT1	CT2	CT3	CT4	CT5
5 cm	22.73	23.70	23.22	22.49	22.27	23.47	24.14	23.86	23.2	22.89
10 cm	21.14	22.06	21.61	20.93	20.72	22.14	22.48	22.19	21.64	21.34
15 cm	19.09	19.91	19.50	18.89	18.71	19.6	20.16	19.86	19.64	19.1
20 cm	19.61	20.45	20.03	19.41	19.21	19.95	20.54	20.72	19.74	19.46
25 cm	19.97	20.83	20.40	19.77	19.57	20.18	20.77	20.41	19.94	19.61

	FT1	FT2	FT3	FT4	FT5	FT1	FT2	FT3	FT4	FT5
5 cm	22.82	22.78	22.63	22.37	21.93	22.80	23.44	23.17	22.53	22.23
10 cm	21.24	21.20	21.06	20.82	20.41	21.5	21.83	21.56	21.01	20.72
15 cm	19.19	19.16	19.00	18.80	18.42	19.03	19.58	19.28	19.07	18.54
20 cm	19.71	19.69	19.59	19.31	18.92	19.37	19.94	20.12	19.17	18.90
25 cm	20.08	20.05	19.97	19.67	19.27	19.6	20.16	19.82	19.36	19.04

	WT1	WT2	WT3	WT4	WT5	WT1	WT2	WT3	WT4	WT5
5 cm	22.73	23.48	22.93	22.43	22.10	22.98	23.56	23.24	22.76	22.39
10 cm	21.13	21.85	21.33	20.88	20.56	21.43	22.43	21.59	21.19	20.84
15 cm	19.14	19.73	19.25	19.85	18.56	19.16	19.6	19.27	19	18.59
20 cm	19.66	20.27	19.81	19.36	19.07	19.49	19.96	19.84	19.26	18.96
25 cm	20.03	20.64	20.19	19.72	19.42	19.70	20.18	19.89	19.48	19.15

C represents control irrigation; W represents wet irrigation; F represents flood irrigation; and T1, T2, T3, T4, and T5 represent the five fertilization treatments.

### Variation in radiation interception

**Figure 3:**
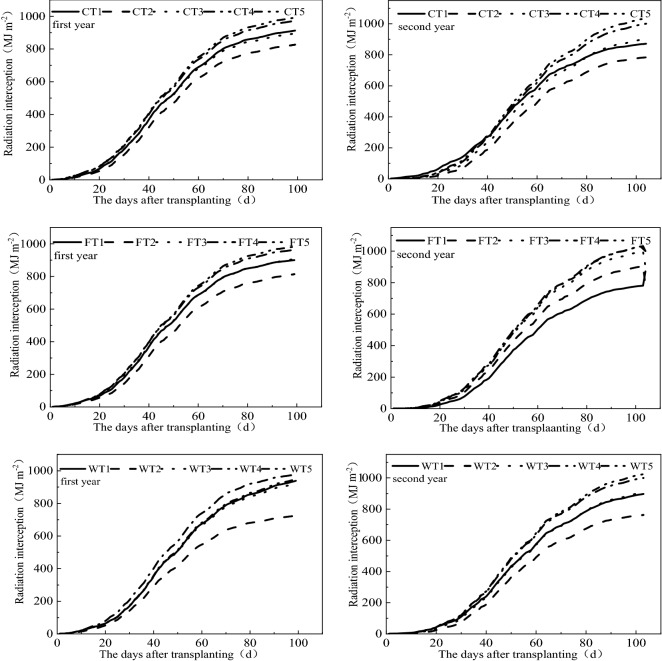
Dynamic changes in the accumulation interception amount in different treatments. Note: C represents control irrigation; W represents wet irrigation; F represents flood irrigation; and T1, T2, T3, T4, and T5 represent the five fertilization treatments.

The variation in radiation interception under the different humic acid fertilizer and irrigation method conditions is shown in Fig. 3. Overall, interception in terms of radiation was mainly completed during three plant growth stages: the noninterception stage (0–20 days), fast interception stage (20–70 days) and slow interception stage (70–100 days). The change in radiation interception was nonlinear over time, and radiation interception differed under the influences of the different humic acid fertilizer and irrigation methods. However, the changing trend of radiation interception under the different treatments was synchronous. Under the different humic acid fertilizer conditions, the greatest radiation interception was obtained in the T5 treatment, and the minimum value was recorded in the T2 treatment. In addition, the radiation interception under control and flood irrigation was greater than that under wet irrigation. As a whole, in the two years, the experiment showed that when humic acid fertilizer was applied to the soil at 1500 kg ha^−1^ under control irrigation conditions, the values of radiation interception were the maximum, 983.3 MJ m^−2^ and 1034.8 MJ m^−2^, respectively.

### Variation in plant height

**Figure 4:**
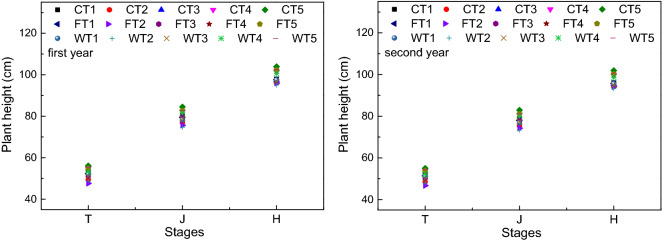
Plant height in different treatments. Note: C represents control irrigation; W represents wet irrigation; F represents flood irrigation; T1, T2, T3, T4, and T5 represent five fertilization treatments; and T, J, and H represent tillering, jointing, and heading, respectively.

The performance in terms of plant height was similar to radiation interception, and gradually increased, as shown in Fig. 4. The main growth in plant height occurred during the jointing stage. Across all humic acid fertilizer and irrigation methods, the plant height was significantly different at the tillering, jointing and heading stages. Moreover, there were significant differences among the gradients under the different humic acid fertilizer and irrigation methods in terms of plant height. According to the results from the tillering stage observation, the plant height in the WT2 treatment was the lowest value in the two growth seasons, while that in the CT5 treatment had the largest value. In the first growth season, compared with the other treatments, the plant height in the CT5 treatment increased by 7.51% (*P* < 0.05), 6.09% (*P* < 0.05) and 5.45% on average at the tillering, jointing and heading stages, respectively, while in the second growth season, the plant height increased by 9.57% (*P* < 0.05), 8.16% (*P* < 0.05) and 7.48% (*P* < 0.05), respectively. The increase in plant height during the two growth seasons was significant.

### Rice fluorescence indices

**Figure 5:**
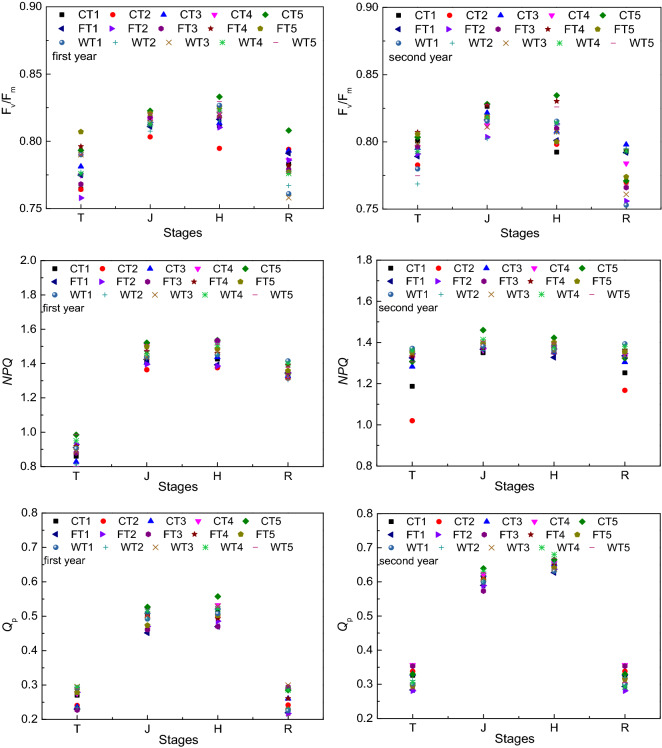
The fluorescence indices in different treatments. Note: C represents control irrigation; W represents wet irrigation; F represents flood irrigation; T1, T2, T3, T4, and T5 represent five fertilization treatments; and T, J, H, and R represent tillering, jointing, heading and ripening, respectively.

The maximum photosynthetic efficiency of PSII (*F*_v_*/F*_m_), nonphotochemical quenching (*NPQ*) and photochemical quenching (*Q*_P_) under different treatments were compared. The differences are shown in Fig. 5. In general, the changes in the fluorescence indices of rice differed under the different humic acid fertilizer and irrigation methods, showing a nonlinear change during the entire growth period. Overall, the *F*_v_*/F*_m_, *NPQ* and *Q*_P_ at the jointing and heading stages were higher than those at the tillering and ripening stages in the two years. In our experiment, the main differences in fluorescence indices occurred at the jointing and heading stages; at these stages, the *F*_v_*/F*_m_ values of CT5, FT5 and WT5 were larger than those of the others, and the best value was recorded in the CT5 treatment. For the other treatments, we did not find statistical significance at the 5% level (*P* > 0.05). The differences in *NPQ* at these two stages were significant. According to the results from the jointing and heading stage observations, the *NPQ* in the CT5 treatment was significantly higher than that in the other treatments (*P* < 0.05). In the first season, compared with that in the other treatments, the *NPQ* in the CT5 treatment increased on average by 6.39% (*P* < 0.05) and 5.94% (*P* < 0.05) at the jointing and heading stages and increased on average by 6.58% (*P* < 0.05) and 4.60%, respectively, in the second season. In general, the *Q*_P_ under control irrigation was greater than that under flood and wet irrigation (*P* < 0.05). Moreover, there were no significant differences among the gradients under the different humic acid fertilizer application methods in terms of *Q*_P_ (*P* > 0.05). Considering the two factors of irrigation and humic acid fertilizer, the main factor affecting the change in *Q*_P_ was the irrigation method, and the effects of the irrigation method were greater than those of humic acid fertilizer.

### SPAD

**Figure 6:**
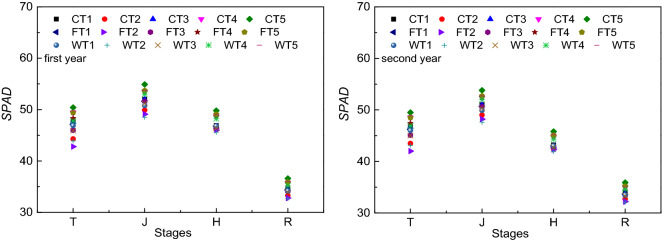
The *SPAD* values in different treatments. Note: C represents control irrigation; W represents wet irrigation; F represents flood irrigation; T1, T2, T3, T4, and T5 represent five fertilization treatments; and T, J, H, and R represent tillering, jointing, heading and ripening, respectively.

The performance in terms of *SPAD* differed from that of fluorescence indices, and there were also similarities, as shown in Fig. 6. In contrast to the fluorescence indices, higher *SPAD* values occurred during the tillering, jointing and heading stages, and the growth process was also nonlinear. The order of the *SPAD* values of the leaves in the different humic acid fertilizer and irrigation methods during different growth stages was as follows: jointing stage > tillering stage > heading stage > ripening stage. The results of the final measurement showed that the *SPAD* values across all humic acid fertilizer and irrigation methods were higher than that in the WT2 treatment. *SPAD* values were higher under the CT5 and FT5 treatments than under the WT2 treatment at the tillering, jointing and heading stages, showing increases of 12.89% (*P* < 0.05) and 9.10% (*P* < 0.05), 11.65% (*P* < 0.05) and 7.82% (*P* < 0.05), and 8.43% (*P* < 0.05) and 5.09%, respectively, in the first season and 12.92% (*P* < 0.05) and 11.13% (*P* < 0.05), 11.58% (*P* < 0.05) and 9.61% (*P* < 0.05), and 8.53% (*P* < 0.05) and 7.07% (*P* < 0.05), respectively, in the second season. Higher increases were achieved in the CT5 treatment.

## Discussion

**Figure 7:**
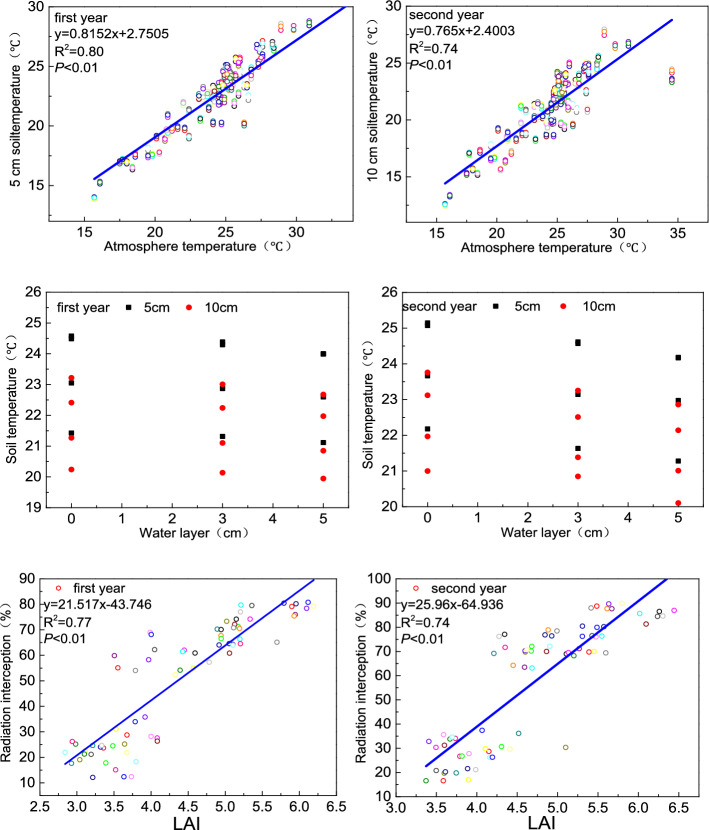
Analysis of influencing factors. Note: *LAI* represents the leaf index.

The different humic acid fertilizers and irrigation methods affected temperature, but not significantly. According to the results, we found that the main factors influencing temperature were the external environment, such as atmospheric temperature. As shown in Fig. 7, the soil temperature was significantly positively correlated with atmospheric temperature, while the water layer was also the main factor that affected the soil temperature. Under the control irrigation conditions, there was no water layer in the field and the specific heat capacity of the soil was small, absorbing heat quickly. With flood and wet irrigation, due to the existence of the water layer, heat absorption was slower than in the soil; therefore, temperature was lower than that with control irrigation. However, due to the different growth statuses of rice among the different humic acid fertilizer treatments, the leaf area index exhibited a large difference, which affected the interception of solar radiation and therefore affected the surface temperature of the soil. However, there was no significant difference among the different treatments.

Because of the differences in the leaf area index, radiation interception also had a significant difference, and in our experiment. The CT5 treatment was higher than the other treatments. Humic acid fertilizer is a plant biostimulator that can promote seed germination^25^, stimulate plant root growth, and promote the growth of aboveground plants^26,27^. Plant height is often regarded as a measure of the photosynthetic and respiratory capacity of plants. Relatively tall plants have more plentiful branches and leaves and thus capture a greater amount of solar radiation^28^. In our experiment, plant height increased with the increase in humic acid fertilizer application, and in both years, the highest value was recorded in the T5 treatment. Among the three irrigation methods, under the control irrigation conditions, the plant height was larger than that under flood and wet irrigation. Humic acid fertilizer can be absorbed by plant roots, and the humic acid fertilizer adsorbed on the cell wall can increase the number of proton pumps on the plasma membrane, which can promote the output of H^+^ to the cell, acidify the cell wall, hydrolyse the polysaccharide, soften and relax the cell wall, and easily elongate and divide the cell, thus promoting plant growth^29^. The growth and development of plants are inseparable from photosynthesis. Chlorophyll and carotenoids are the main pigments of leaf photosynthesis, and they are the basis for plants to absorb, transmit and convert light energy^30^. Studies have shown that humic acid fertilizer could significantly improve the *SPAD* of pepper^31^, wheat^32^, and maize^33^, and the results were similar to our experiment. In our study, from the tillering to ripening stages, the highest value of *SPAD* was in the T5 treatment, and under different irrigation conditions, control irrigation was the best. When humic acid fertilizer coupled with the irrigation method was added to the soil, the fluorescence parameters increased significantly, which indicated that the photosynthetic apparatus of leaves was healthy and had good function. Therefore, it had a higher opening degree and excitation energy capture efficiency; at the same time, the excitation energy of the reaction centre increased promoting the formation of plant photosynthetic capacity.

## Conclusion

During the two-year experiment, we studied the effects of different humic acid fertilizers and irrigation methods on farmland soil temperature, radiation and rice growth. The main results could be summarized as follows: (1) Across all treatments, temperature was not significant, while the surface soil temperature was higher than that in the other soil layers. (2) The change in radiation interception was nonlinear over time, and radiation interception differed under the influences of the different humic acid fertilizer and irrigation methods. However, the changing trend of radiation interception under the different treatments was synchronous. (3) At the jointing and heading stages, the *F*_v_*/F*_m_ values of CT5, FT5 and WT5 were larger than those of the other treatments. The differences in *NPQ* at these two stages were significant, and that in the CT5 treatment was significantly higher than that in the other treatments. In general, the *Q*_P_ under control irrigation was greater than that under flood and wet irrigation. Moreover, there were no significant differences among the gradients under humic acid fertilizer conditions. Additionally, *SPAD* values were higher under the CT5 and FT5 treatments. Overall, the humic acid fertilizer and irrigation method could affect the soil temperature, radiation and rice growth, and we found that the CT5 treatment was better. It is feasible to completely replace chemical nitrogen fertilizer with humic acid fertilizer in the short term; however, it is impossible for crops to obtain higher yields without chemical nitrogen fertilizer for a long time. Therefore, to provide a basis for scientific fertilization, we should further verify the year in which the yield decreases significantly under the condition of humic acid fertilizer completely replacing chemical nitrogen fertilizer.

## Data Availability

The datasets used and/or analysed during the current study are available from the corresponding author on reasonable request.
